# Exchange-Traded Funds on European Markets: Has Critical Mass been Reached? Implications for Financial Systems

**DOI:** 10.3390/e22060686

**Published:** 2020-06-19

**Authors:** Adam Marszk, Ewa Lechman

**Affiliations:** Faculty of Management and Economics, Gdansk University of Technology, Narutowicza 11/12, 80-233 Gdansk, Poland; eda@zie.pg.gda.pl

**Keywords:** diffusion, financial innovations, critical mass, exchange-traded funds, investment funds

## Abstract

Exchange-traded funds (ETFs) are one of the most rapidly expanding categories of financial products in Europe. One of the key yet still unanswered questions is whether European ETF markets have reached the size at which they could affect the financial systems. In our study, we examine 13 European countries during the period 2004–2017 in order to trace whether the share of ETFs in the total assets of investment funds has reached the ‘critical’ level that makes possible their further growth and can be associated with an influence on the financial system. We use a novel methodological approach that identifies the ‘critical mass’ along diffusion trajectories. Our results show that, in 10 countries, the share of ETFs in assets of investment funds increased. Still, in most countries, the share of ETFs did not exceed 1%. Estimates of the diffusion models indicate that the process of growing shares of ETFs was most dynamic and relatively most stable in Switzerland and United Kingdom. Results of the critical mass analysis imply that its achievement may be forecasted exclusively in these two cases. However, even in such cases there is no substantial evidence for a possible significant influence of ETFs on the local financial systems.

## 1. Introduction

Investment funds are a vital part of the modern financial system as they participate in operations made in other segments of the financial sector such as capital markets or banking. Over the last three decades, the investment funds industry has experienced substantial changes resulting from, among other factors, the adoption of new technologies in the financial system. Another partially linked development was the launch and surging popularity of new types of funds, among them exchange-traded funds (ETFs) as the most notable example (of course, there are considerable differences between countries and regions).

Referring to the explanation of the similar concept (‘investment company’) used by the US Investment Company Institute [1], investment funds may be defined as corporations that invest their shareholder’s funds in securities or other assets in line with the organization’s objective. There are many types of investment funds, with classifications based on various criteria. In our research, we analyze two of the basic categories: first, mutual funds, as the conventional type of investment funds, well-established in the financial system, and second, a much newer alternative, ETFs, which, for reasons outlined in Section 2, may be regarded as an example of innovations in the financial system. 

In our research, we focus on the European investment funds industry for two main reasons. First, it is one of the most developed in the world as, in terms of assets, as of late 2018, the combined European market accounted for ca. 40% of the global investment funds’ assets, only behind the markets in North and South America (mostly in the United States, which is the global leader in the investment funds industry) whose share was at approximately 50% [2]. The share of the European ETFs in the global assets of such funds was, however, much lower, at only 16% (due to much more significant prevalence of the US funds). The second reason is the substantial diversity of the ETF markets in the European region as it includes both large ETF markets, with considerable assets (e.g., Switzerland) and countries which clearly lag behind, with nascent ETF markets (e.g., Hungary or Poland). This variety allows for a comparison of ETF adoption in countries where macroeconomic conditions and financial systems are to large extent shaped by common factors, resulting from, for example, EU membership or strong economic links to this organization.

The aim of this study is to evaluate the diffusion trajectories of ETFs in the European countries, with the special focus on the identification of the ‘critical mass’. More specifically, our aim is to trace whether the share of assets of ETFs in the total assets of investment funds has reached the ‘critical’ level that makes possible their further expansion in the financial system. Additionally, we discuss whether, at the level of diffusion reached in the European countries, ETFs may be considered to affect their financial systems, either positively or negatively. Our empirical framework encompasses presentation of the basic facts about the assets of the European investment funds with the descriptive statistics albeit the key research method are diffusion models, used to assess the diffusion path of ETFs in Europe and to identify their critical mass. Our analysis covers 13 European countries: France, Germany, Greece, Hungary, Ireland, Italy, Norway, Poland, Spain, Sweden, Switzerland, Turkey, and the United Kingdom. Quarterly data on the assets of investment funds were extracted from the datasets of the European Fund and Asset Management Association and Lipper.

This paper contributes to the present state of knowledge in two ways. It is the first research based on quarterly data on the assets ETFs (previously annual data or other indicators were applied yet using quarterly data facilitates more detailed examination of the developments in the investment funds industry) and, even more importantly, it shows the application of the critical mass framework to study one part of the financial sector, namely the investment company industry. Diffusion (spread of innovations) models were previously rarely applied to analyze the investment funds (only a few such papers had been published (e.g., [3,4]). There are, however, relatively many papers on the diffusion of other types of financial innovations (see, e.g., [5,6,7,8,9]). There were no previous attempts to study the critical mass in the adoption of innovations in the financial system, despite potentially significant conclusions that may be reached about the possible influence of ETFs on the financial sector.

The paper is divided into five sections, beginning with the introduction. The second section presents the basic features of ETFs, compares them to the mutual funds, and discusses their denotation as financial innovations and the potential impact on financial systems. The third section outlines data sources and the empirical setting. Section 4 is divided into three parts. The first part includes preliminary evidence on the assets of investment funds in Europe. The second part discusses the empirical results obtained with the application of diffusion models. The third, major part of this section outlines results of the critical mass analysis with reference to the diffusion of ETFs, including evaluation of their possible impact on the financial system in the countries in the scope of our research. The paper concludes with the fifth section.

The paper is a substantially expanded version of the paper entitled “Exchange-traded funds in Europe: diffusion in investment funds industries” that was presented during 10th International Conference on Currency, Banking and International Finance.

## 2. ETFs in the Financial System: Selected Theoretical Issues and Literature Review

This section begins with the presentation of the main features of ETFs. Next, they are compared to the mutual funds. We also present the selected concepts of their inclusion in the financial innovations category. Finally, we discuss their possible role in the financial systems, both positive and negative.

### 2.1. ETFs Versus Mutual Funds. ETFs as Financial Innovations

Exchange-traded funds (ETFs) represent one of the most recent types of investment product offered in the financial sector (in comparison to other categories of investment funds whose history is much longer) and the first such funds were launched in Canada and the United States in the early 1990s [10]. ETFs may be defined as investment funds whose shares (units) are traded on stock exchanges or similar trading venues at market-determined prices, analogously to, for example, listed stocks or fixed income securities [1]. From the perspective of the investor, it means that ETF market may be accessed through, for example, a brokerage account. 

The objective of the first ETFs available to investors was to track (follow) the returns on their benchmarks, in that case defined as selected equity market indexes, minimizing any possible tracking errors. ETFs were, therefore, a passive type of investment fund. Passive equity ETFs are still the largest category in all possible dimensions of the market development (assets, turnover, number of funds etc.). However, over the years the global ETF industry has become much more diversified and currently many different types of ETFs are available, with various investment aims in relation to their benchmark (e.g., semi-passive), modified returns (e.g., leveraged) and, above all, with exposure to various types of assets—not only equities but also bonds, commodities and other [11,12,13,14]. As a result, it may be stated that ETFs have grown to be an alternative for most categories of other, more established investment funds, not necessarily merely passive equity funds. 

One fundamental feature of ETFs is the duality of the transactions in their shares which occur on two basic types of markets: primary and secondary [15,16,17]. On the primary market, shares of ETFs are created and redeemed in the course of transactions between the fund (more technically: the fund’s provider) and authorized participants. Investors who wish to buy or sell the shares of ETFs conduct such operations on the secondary market (i.e., not directly with the fund’s provider as in the case of mutual funds—see the definition of mutual funds below). Second, unique (in the investment funds industry) fundamental attribute of ETFs is the creation and redemption process of their shares (which takes place on the primary market). This is labeled as ‘in-kind’ because it involves an exchange of fund’s shares for the securities or other assets held by the fund, with the limited use of cash [16]. In the case of passive funds, ‘in-kind’ mechanism facilitates arbitrage transactions which limit tracking errors. It must be underlined that in-kind creation and redemption is used exclusively in the case of funds which manage the portfolio of the securities that constitute their benchmark (in some cases using various optimization techniques [12]) and such funds are labeled ‘physical’ ETFs. Funds which employ derivatives in order to gain the declared exposure (usually total return swaps) are called ‘synthetic’ and the creation and redemption of their shares is conducted with cash.

In contrast with ETFs, mutual funds may be considered to be a much more broadly recognized type of investment fund; on a global scale they are the largest category group in terms of assets or number of products offered by financial corporations. Moreover, in many countries they are the only category available for local investors. There are some discrepancies between various regions with regard to the terms used to label this group. We use ‘mutual funds’ throughout the paper as it underlines clearly the distinction between this category and ETFs. The other terms, such as ‘open-end funds’, despite their broad usage, can blur the main differences (in fact both ETFs and mutual funds have the open-end structure—see discussion below) while further ones, such as ‘unit trusts’ are too country-specific and/or focused on the legal aspects. In our research we utilize the definition of mutual funds provided by The International Investment Funds Association (IIFA; Toronto, Canada) which defines them as an investment company (we use the term investment fund) that buys a portfolio of assets (usually securities) in order to meet a specified investment objective; mutual fund is ready to buy back (redeem) its shares on demand by investor; the issuance of new shares is continuous in most mutual funds [18].

In order to fully understand the advantages offered by ETFs in comparison to the mutual funds, it is necessary to present a comparison of their attributes. The main differences, which may be regarded as relative advantages of ETFs, are summarized in Table 1. Another benefit of ETFs, although highly fund-specific, is lower costs for investors in most cases [11]. Among the similarities, it should be underlined that both categories have the open-end structure which means that the number of their shares is not fixed (as in closed-end funds) and it can change due to their creation and redemption. Moreover, they give the holders of their units access to similar investment strategies (e.g., exposure to equities) and are managed by financial institutions [12].

For the reasons presented in the preceding paragraphs, ETFs may be regarded as a category of investment funds with applications similar to the conventional, well-established group, namely mutual funds. However, their features (e.g., higher transparency) make them more beneficial for many users. Consequently, ETFs may be considered to be an example of financial innovations. Another rather obvious reason for such denotation of ETFs is their much shorter history yet almost thirty years in case of the oldest North American markets may be perceived as rather long period, especially bearing in mind the high dynamics of the financial system. In order to more fully justify our approach to the analysis of the ETF markets, which requires assuming that they are innovative in comparison to other investment funds, we refer to the selected (only a few, for brevity we chose some of the most often cited publications in the field of ETFs) publications whish use such a designation (see Table 2). As it may be noticed, despite differing approaches to the exact definition of ETFs (and their place in the financial system), even in the most recent publications, ETFs are designated as financial innovations.

### 2.2. Role of ETFs in the Financial System: Potential Positive and Negative Effects of the Diffusion of ETFs

Growing assets of ETFs in many countries have resulted in the recent years in intensification of the discussion concerning their possible positive and negative impact on the financial system. Probably the most frequently analyzed event in the global economy during the early years of the XXI century is the 2008 financial crisis. Nonetheless, the size of the ETF markets, even the biggest ones such as the United States, had been rather small up to 2008 so the possible influence of ETFs on the emergence and spread of the financial turmoil was marginal. However, since 2008, ETF markets have grown rapidly in some of the most advanced countries, such as the USA, Japan, or the United Kingdom. This has led to a growing interest of researchers as well as various supervisory national and international institutions in the possible consequences of the broader adoption of innovative funds. This attention was magnified by various changes in the ETF industry (e.g., increasing application of synthetic (derivatives-based) fund structures in Europe—their popularity peaked in 2008–2010) or events such as the first and second Flash Crashes on the US capital markets in 2010 and 2015 [13], with a substantial role played by ETFs.

In Table 3, we summarize the selected key areas of the possible negative impact of the diffusion of ETFs on various elements of financial systems. We considered both the impact on the market participants (particularly the users of ETFs) and the linked assets, and broader consequences concerning entire markets. Positive effects of the ETFs diffusion have attracted much less attention of researchers and are rarely discussed; they include:

- extension of the group of investors accessing financial markets, attracted by the features of ETFs such as their diversity or other benefits in relation to the conventional funds (see details in Table 1);
- increased liquidity of the securities whose prices are tracked by passive ETFs due to, above all, improved price discovery mechanism [13,27,28,29,30] or relaxation of the short-sale constraints [31]. However, there are also studies that provide evidence for the opposite effect, i.e., decreased liquidity (e.g., [23,32,33]);
- potential positive effects of the greater financial openness stemming from the development of cross-listed ETFs or funds offering exposure to foreign assets [34].

To supplement the discussion of the possible influence of ETFs on the financial systems, and to justify our focus on ETF markets reaching the ‘critical mass’, it must be added that the minimum level of the size of ETF market, necessary for the emergence of either positive or negative outcomes, has still not been recognized. Nevertheless, the frequent assumption is that the probability of emergence of potential benefits or threats will increase with the increasing size of the ETF market (see, for example, [38,44]). However, this assumption was not verified empirically—even though there are a number of studies that examined the relationships between ETFs and particular linked assets (e.g., equities within the tracked stock indexes (see, e.g., [45,46,47]), including studies concerning the European ETFs, there were no previous attempts to check if the size of the local ETFs markets has reached the level that substantiates stating that they may exert sustainable influence on a broader scale. 

## 3. Materials and Methods

This section outlines the sources of data and methodological setting of our research. First, we present the details of our dataset, including the applied indicators and time period of the analysis. Second, we discuss the research methods: diffusion model and critical mass framework. 

### 3.1. Data Sources

Our analysis covers 13 European countries with ETFs listed on local exchanges (in alphabetical order): France, Germany, Greece, Hungary, Ireland, Italy, Norway, Poland, Spain, Sweden, Switzerland, Turkey, and United Kingdom. It means that we evaluate most European ETF markets. We do not study the markets with complicated structure which hinders their evaluation such as Baltic states with joint stock exchange or Luxembourg where many ETFs are domiciled but only a few are actually traded. Another exception is the Russian Federation due to lack of data on the investment funds consistent with data obtained for other countries; the size of the Russian ETF market is, though, rather small. Additional explanation is required for France where the stock exchange in Paris is part of the Euronext Group. Hence, due to the prevalence of the French segment, we classify all ETFs on Euronext as part of the French ETF market. 

A core indicator applied to reach the aims of our research is net assets (assets minus liabilities) of investment funds in the selected European countries (henceforth: ‘assets’), expressed in USD millions for comparability purposes. Here, we use end-of-quarter values. ‘Total assets’ are aggregated assets in particular country. In the case of ETFs, we use data on the funds which are primary listed (and traded) on the stock exchanges in the particular country—this serves as the basis for our classification (due to possible inconsistencies between data for investment funds and ETFs we could not use the classification based on the physical location of the assets held by ETFs; another reason was data availability). ‘Investment funds’ are all categories of funds included in this category by the data providers (see below), above all, various types of mutual funds and ETFs.

The database used in this research is combined from two datasets. The first dataset includes quarterly observations of the total market for investment funds extracted from the statistical releases of the European Fund and Asset Management Association’s (EFAMA). The second dataset, which covers exclusively ETFs, is derived from the Lipper database provided by Refinitiv (formerly Thomson Reuters). Country-level quarterly estimates were calculated based on individual funds’ data (as of end of each consecutive quarter). Our database covers more than two thousand European ETFs. All individual observations were verified (by checking for missing data or inputting errors). To the best of our knowledge, it is one of the first research on the European investment funds (and ETFs in particular) in which fund-level observations were aggregated into country-level indicators.

The time span of the analysis is subject to data availability, as a dataset on the assets of investment funds was acquirable for the period from second quarter of 2004 to the third quarter of 2017, i.e., 54 consecutive quarters. Moreover, in most countries in our sample, there were no ETFs before 2004.

### 3.2. Empirical Setting (Diffusion and Critical Mass)

In our research, referring to the widespread perception in the relevant literature (see Section 2.1), we claim that ETFs may be regarded as innovations in the financial system. Moreover, to substantiate this claim, we can refer to some of the broadly used concepts of financial innovations. For instance, according to one of the most popular definitions suggested by Allen and Gale [48], they are securities with some novel attributes. ETFs are highly consistent with this concept as they are both securities and provide users with novel features in relation to conventional investment funds. The other yet to some extent similar definition was presented by Lerner and Tufano [49] who stressed that financial innovation should be considered as the introduction and development of new instruments in the financial system; ETFs can be regarded as such instruments—even though in some countries they were launched several years ago, their development is still taking place (moreover, there are still many countries without ETF markets). Finally, ETFs can be included in the classification and definition of financial innovations outlined in Frame and White [50] because they are new financial products which in some ways more fully satisfy the needs of their users than the conventional products.

It may be stated, in the context of the theoretical concepts of the diffusion, that gradually increasing adoption of ETFs among investors can occur due to ‘word of mouth’ and emerging network effects [51]. We assume that investors who use these innovative funds may freely contact users of other types of investment funds and people or institutions that previously did not use any category of such financial products. In short, we assume that diffusion of ETFs takes place on the European markets for investment funds which may be observed in two dimensions: the increasing total assets of ETFs as well as their growing share in the total assets of investment funds. The latter is our main approach—henceforth, unless stated otherwise, diffusion of ETFs is measured in terms of their shares.

Bearing in mind the above-mentioned clarifications, and to achieve the main research goals of this work, we use a methodological framework that allows identifying the process of diffusion of ETFs. Therefore, we use not only elementary descriptive statistics but also innovation diffusion models (see for instance, [51,52,53,54]), which appear to be an appropriate tool to approximate ETF diffusion trajectories. An analogous approach to the identification of ETF market development patterns can be traced in the study by Lechman and Marszk [4] in which ETF diffusion paths were analyzed across selected countries. 

As argued in our research, we use the innovation diffusion model. Mansfield [55] and Dosi and Nelson [56] analyzed the phenomenon of the process of diffusion of innovation by adopting the evolutionary dynamics concept, which may be well approximated by the logistic growth function. The logistic growth function can be presented as following ordinary differential equation [57]:

(1) $$\frac{dY_{x}\left(t\right)}{dt}=\alpha \;N_{x}\left(t\right),$$

where N(t) is the level of variable x, t is time, and α is the constant growth rate. 

The model in Equation (1) is pre-defined as exponential, without growth limits imposed, which potentially may lead to unrealistic projections, as different systems are usually constrained and hence do not grow infinitely. To challenge the latter, we add the ‘resistance’ parameter in Equation (1), imposing an upper ‘limit’ to the logistic growth [53], which automatically changes the shape of the growth curve, making it sigmoid—S-shaped. 

The modified version of Equation (1) follows:

(2) $$\frac{dY\left(t\right)}{dt}=\alpha N\left(t\right)\left(1-\frac{N\left(t\right)}{\kappa }\right),$$

where *κ* is the imposed upper asymptote that limits the growth of Y(t). 

The 3-parameter logistic differential equation can be re-written as a logistic growth function that takes only non-negative values throughout its whole path:

(3) $$N_{x}\left(t\right)=\frac{\kappa }{1+\exp\left(-\alpha \left(t-\beta \right)\right)\;},$$

where $N_{x}\left(t\right)$ denotes the value of variable x in time period t. The parameters (The parameters in Equation (3) can be estimated using, for instance, ordinary least squares (OLS), maximum likelihood (MLE), algebraic estimation (AE), or nonlinear least squares (NLS). According to Satoh [58], NLS yields relatively best predictions—the estimates of standard errors are more valid than in other methods. Moreover, using NLS allows avoiding time-interval biases, which are problematic in the case of OLS [59]. The key disadvantage of NLS is sensitivity of the parameters to the initial values in the time-series.) in Equation (3) can be interpreted as:

*κ*—upper asymptote that determines the limit of growth;
α—growth rate that determines the speed of diffusion;
β—midpoint that determines the exact time ($T_{m}$) when x reaches 0.5κ; it indicates the inflection point of the logistic curve.

To facilitate interpretation of the diffusion pattern, a ‘specific duration’ parameter that shows the time needed for x to grow from 10%*κ* to 90%*κ*. 

In our research we use the Equation (3) to estimate the parameters of country-specific patterns of diffusion of ETFs, and with this aim we define:

(4) $$ETF_{i}\left(t\right)=\frac{\kappa_{i}^{ETF}}{1+\exp\left(-\alpha_{i}^{ETF}\left(t-\beta_{i}^{ETF}\right)\right)\;},$$

where i denotes country. 

Moreover, in our research, we assume that the process of the diffusion of ETFs is to a large extent analogous to the process of diffusion of innovations (especially technological innovations). Hence, to enrich this study we use the novel methodological approach developed and presented in Lechman [54,57] that allows identification of the ‘critical mass’ along diffusion trajectory. According to Lechman [54], ‘critical mass’ (For the notion of the ‘critical mass’, see the works of Cabral [60,61], Marwell and Oliver [62], Economides and Himmelberg [63,64], Molina et al. [65], Rogers [52], Evans and Schmalensee [66], or Puumalainen et al. [67].) stands for the minimal and necessary number of users of new technology that ensures the emergence of the ‘take-off’ along the diffusion trajectory, and at which the further process of diffusion becomes self-perpetuating. Critical mass defines a unique threshold that preconditions the emergence of the take-off along the diffusion pattern and gives rise to logistic growth of examined variable. 

To calculate critical mass, first we define the replication coefficient $\Phi_{i,t}$ and marginal growth $\Omega_{i,t}$ of the analyzed variable over time, where i denotes country and t year. These calculations rely on time series analysis of given variable. We account for period-to-period changes in the value of examined variable—this is like chain changes, and we confront them with the absolute growths of the variable.

If for a given country i $N_{i,t}$ is the level of technology adoption in t year, then the replication coefficient is defined as:

(5) $$\Phi_{i,y}=\frac{N_{i,t}}{N_{i,t-1}}.$$

Referring to Equation (5), if $N_{i,t}>0$ and $N_{i,t-1}>0$, $\Phi_{i,t}\;\in \left(0;\infty \right)$ explains the multiplication of technology users that exhibits the period-to-period dynamics of the diffusion process. For $\Phi_{i,t}>1$ the number of users of new technology increases; for $\Phi_{i,t}=1$, the number of new technology users is constant over time; for $\Phi_{i,t}<1$ the number of users of new technology decreases over time. Hence, intuitive analysis of the $\Phi_{i,y}$ shows how fast the process of diffusion proceeds in each consecutive time period. This gives much broader picture of in-time dynamics of the process than could be derived for intrinsic growth rate returned from logistic growth estimations. Lechman [54] discussed also the concept of ‘marginal’ growth in technology adoption $\Omega_{i,t}$:

(6) $$\Omega_{i,t}=N_{i,t}-N_{i,t-1},$$

under the conditions that $N_{i,t}>0$ and $N_{i,t-1}>0$. The value of $\Omega_{i,t}$ indicates the change in the total number of users of new technology over two consecutive years.

Two defined coefficients—$\Phi_{i,t}\;$and $\Omega_{i,t}$ are closely interrelated, and these relationships may be expressed as:

(7) $$\Omega_{i,t}=N_{\left(i,t-1\right)}\left[\Phi_{i,t}-1\right]$$

where $\Omega_{i,t}$ depends on the dynamics of the replication process, and examining the $\Phi_{i,t}$ and $\Omega_{i,t}$ simultaneously, we observe that (Lechman, 2015):

1. If $\Phi_{i,t}>1$, then $\Omega_{i,t}>0$ the replication process is sufficiently strong and the diffusion proceeds, which is demonstrated by the increasing number of new users;
2. If $\Phi_{i,t}=1$, then $\Omega_{i,t}=0$: no replication is reported and the diffusion does not proceed, which results in a constant number of users;
3. If $\Phi_{i,t}<1$, then $\Omega_{i,t}<0$: the replication process is so weak that the diffusion is limited, and there will be a decreasing number of users.

**Figure 1:**
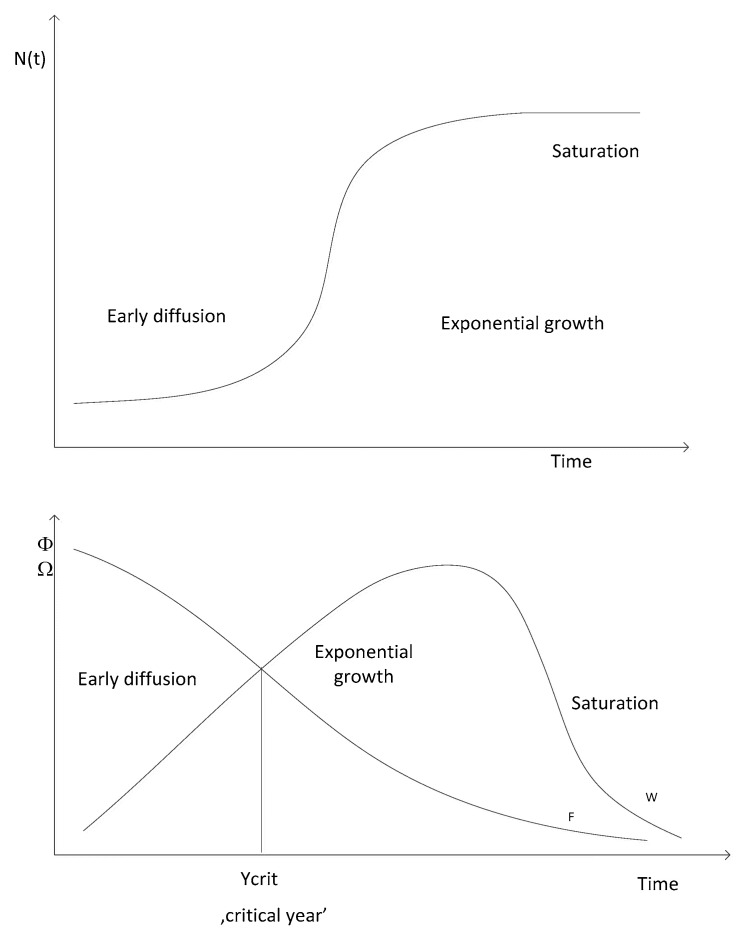
Relationship between the replication coefficient ($\Phi_{i,t}$), and marginal growth ($\Omega_{i,t}$), along the S-shaped diffusion trajectory. Adapted from [54].

Determining ‘critical mass’ is possible when observing the in-time behaviour of respective coefficients—$\Phi_{i,t}$ and $\Omega_{i,t}$—along the sigmoid technology diffusion pattern (see Figure 1).

Initially, during the early diffusion phase, when the growth rates are low, replication coefficient tends to be higher than marginal growth $\left(\Phi_{i,t}>\;\Omega_{i,t}\right)$ and thus a gap emerges between $\Phi_{i,t}\;\mathrm{and}\;\Omega_{i,t}$. This is a natural effect of the early diffusion phase along logistic growth curve, during which initial values and absolute growths (changes) of values of examined variable are relatively low, while the growth rate is high. However, as the variable’s value increases, the period-to-period absolute changes gradually increase, while the growth rates slow down. Hence, as the diffusion proceeds and the replication process gains strength (so that $\Phi_{i,t}>1$ and $\Omega_{i,t}>0$), $\Omega_{i,y}$ ultimately gradually increases while ${}_{i,t}$ decreases over consecutive years, which will inevitably lead to decreasing gap between $\Phi_{i,t}\;\mathrm{and}\;\Omega_{i,t}$ (the paths that show the changes in $\Phi_{i,t}\;\mathrm{and}\;\Omega_{i,t}$ are converging; see Figure 1). If the latter is satisfied, the paths that show changes in $\Phi_{i,y}\;\mathrm{and}\;\Omega_{i,t}$ finally intersect (the gap between $\Phi_{i,t}\;\mathrm{and}\;\Omega_{i,t}\;$is closed). In the next years the replication coefficients are lower than marginal growth $\left(\Phi_{i,t}<\Omega_{i,t}\right)$, and the paths that show changes in $\Phi_{i,t}\;\mathrm{and}\;\Omega_{i,t}\;$diverge. 

If $\Phi_{i,t}=\Omega_{i,t}$ occurs (i.e., intersection of $\Phi_{i,t}\;\mathrm{and}\;\Omega_{i,t}$ on Figure 1) Following this procedure would yield rigid identification of the exact date when $\Phi_{i,t}=\Omega_{i,t}$, which would require using daily data, which for obvious reasons is scarcely possible. To challenge this obstacle, we consider that the critical mass is reached during the first time period when $\Phi_{i,t}<\Omega_{i,t}$, if in the previous quarter $\Phi_{i,t-1}>\;\Omega_{i,t-1}$) then critical mass is achieved, and this inevitably should lead to logistic growth of examined variable over time. Once the critical mass is achieved, we observe that the process of diffusion leaves the initial growth phase (when increases of variable value are slow) and enters fast-growing phase—the logistic growth phase during which we observe rapidly growing variable values. Identification of the exact time period when $\Phi_{i,t}=\Omega_{i,t}$ is based on an empirical analysis of the times series of a given variable, and it allows for the conclusion that when the dynamics of the process of diffusion radically change, it switches from the slow growth to fast (logistic) growth. That change in the in-time process dynamics signals that the process of diffusion has left the initial growth phase along the logistic growth trajectory, and entered the fast growth phase, hence the critical mass (the critical threshold) of the variable value has been achieved. Reaching the critical mass enhances the chain reaction (specific network effect), due to which we observe fast growing values of the examined variable.

If critical mass is not reported, this means that during the early diffusion phase, the replication lacked the strength to ensure gradual increases in $\Omega_{i,t}$ that would lead to closing of the gap between $\Phi_{i,t}\;and\;\Omega_{i,t}$. As result, the patterns showing in $\Phi_{i,t}\;\mathrm{and}\;\Omega_{i,t}$ would diverge. If $\Phi_{i,t}=1$ or $\Phi_{i,t}<1$ (see the discussion in the preceding paragraphs), the situation is similar, and the technology diffusion is impeded. 

Profound analysis of $\Phi_{i,t}\;\mathrm{and}\;\Omega_{i,t}$ paths may be claimed as an alternative for examining the diffusion trajectories and reaching of the critical mass to classical analysis using logistic growth model and curve. 

In our research, we use the above-presented method to identify the critical mass along the trajectory of the diffusion of ETFs. Our aim is to trace whether, across examined economies, the ETFs share in total net assets of investment funds has reached the ‘critical’ level that ensures their further expansion in the investment industry.

## 4. Empirical Results

The main topic of the current section is the presentation of the results of our study. We begin with the discussion of the preliminary evidence on the assets of investment funds in Europe. In the second part we present the empirical results obtained with the application of diffusion models. In the third and final section we outline the results of the critical mass analysis, accompanied by an evaluation of the possible impact of ETFs on the financial system in the countries in scope of our research.

### 4.1. Assets of Investment Funds in the European Countries—Basic Facts

At the end of the third quarter of 2017, the combined assets of investment funds in the 13 analyzed countries reached more than $11 trillion, i.e., the highest value in the history (own calculations based on data extracted from EFAMA reports). Assets of ETFs were also at the historically highest level ($690 billion according to Lipper database) yet they constituted only approximately 6.2% of the entire investment industry. It was, though, the maximum market share of the innovative funds (for comparison, in the United States or Japan it exceeded 10% in 2017). However, examination of the country-level summary statistics shows very deep differences between the analyzed European markets, in terms of both total assets of investment funds or ETFs and shares of the innovative funds (see Table 4).

**Figure 2:**
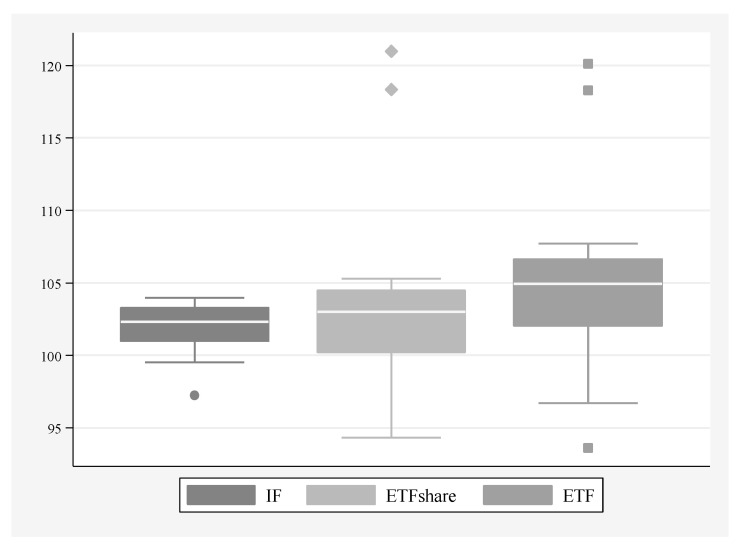
Average quarterly dynamic of the following indicators: total assets of investment funds, share of ETFs in total assets of investment funds, and total assets of ETFs. Period 2004–2017. Authors’ elaboration. Note: IF—total assets of investment funds; ETFshare—share of ETFs in total assets of investment funds; ETF—total assets of ETFs.

The four largest European markets for investment funds (in terms of mean as well as minimum and maximum values) are France, Germany, Ireland and the United Kingdom. For comparison, the four smallest are Greece, Hungary, Poland, and Turkey, and the inclusion of the two last countries in that group proves that the assets of investment funds are not necessarily fully explained by the size of the local economy. In the dynamic perspective, i.e., when observed changes are considered, it may be noticed that only in case of two countries total growth rate was negative, namely Greece and, to lesser extent, Italy (see Figure 2 which proves clearly that Greece may be considered an outlier in our sample if this dimension is considered). Among the countries with a positive growth rate, the lowest one was observed for Spain. Poor results of those three countries (Greece, Italy, and Spain), noticeable in particular between 2008 and 2012, may be linked to the global financial crisis and the eurozone debt crisis which affected them stronger than other economies in our sample. Assets of investment funds grew most quickly in countries with relatively lower starting values (a ‘catching-up’ process), with the notable exception of Switzerland.

Three of the four largest investment funds markets are also the countries with the biggest ETF markets in Europe: France, Germany and the United Kingdom. The only exception is Ireland. Most ETFs domiciled in Ireland are not listed and traded on the stock exchange in that country. If the share of ETFs is considered, Switzerland is the leading country in the region, with the mean value of 7.69%; assets of ETFs in Switzerland are also substantial. Taking into account the average total assets of ETFs, Spain and Sweden may be regarded as mid-sized markets while the remaining countries can be perceived as small ETF markets. Taking into consideration the shares of ETFs in the total markets for investment funds slightly changes these conclusions. Sweden is the only country outside the group of the largest European ETF markets with the share of ETFs exceeding 1%, in the remaining it is significantly lower.

The largest category of ETFs in all European countries is equity ETFs; in the vast majority these ETFs are passive funds which track the returns of the broad market or blue-chip indexes. The share of equity ETFs in the total assets of European ETFs exceeded 70% in 2017, while the share of bond ETFs was approximately 25%, and the role of the remaining categories was marginal. The same applies to the most popular ETFs in particular countries (i.e., in terms of assets). According to the 2017 data some of the largest ETFs which are primary listed in the examined countries included ‘iShares Core S & P 500 UCITS ETF USD (Acc)’ (the largest fund in the United Kingdom), ‘iShares EURO STOXX 50 UCITS (DE) ETF’ (the largest in Germany), ‘Lyxor Euro Stoxx 50 (DR) UCITS ETF D-EUR‘ (the biggest in France), ‘iShares Core EURO STOXX 50 UCITS ETF EUR (Acc)’ (the leader of the Swiss market), and ‘Lyxor FTSE Italia Mid Cap PIR (DR) UCITS ETF D-EUR’ (the biggest in Italy). Generally, the funds with most assets track the US, regional or local equity market indexes. For a more detailed discussion about the structure of the European ETF markets, see [68].

**Figure A1:**
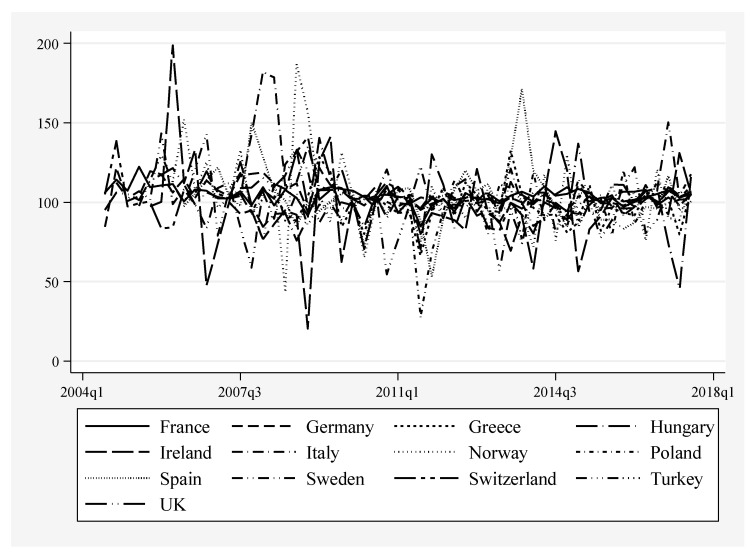
Average quarterly dynamics of the share of ETFs in total assets of investment funds—time lines for 2004–2017 (extremes—excluded). Authors’ elaboration (data sources described in the Section 3.1).

Analysis of the dynamic indicators regarding the European markets shows that in all but three countries the assets or shares of ETFs have increased since 2004 (or since their launch, whichever occurred later). The three exceptions are Greece, Hungary and Poland. In terms of the ETFs’ share, the decline in Poland was most substantial. The strongest increases in the values of assets can be noticed for Italy and Spain (in terms of market shares for Italy and the United Kingdom, the quarterly dynamics of those countries can be noticed as outliers on Figure 2). What is important, all four countries with the largest assets or shares of ETFs have experienced a very strong upsurge which further indicates the substantial diversity of the European investment funds industry, with a small group of distinctive leaders. Figure A1 (in the Appendix A) indicates that highest quarterly dynamic of the ETFs’ share could be observed in the period of the 2008 global financial crisis.

### 4.2. Diffusion of ETFs on the European Markets: Estimates of the Diffusion Models

Descriptive statistics discussed in the preceding Section show the substantial diversity of the European ETF markets. In the current section, we briefly present the estimates of the models of ETFs diffusion; we measure the level of diffusion using the share of ETFs in total assets of investment funds in a particular country. 

**Figure 3:**
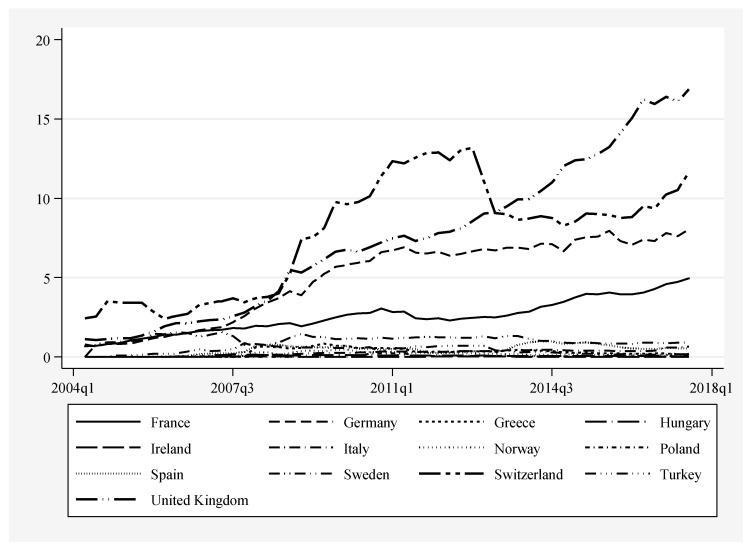
Patterns of ETFs diffusion—cross-country comparisons. Period 2004–2017, quarterly data. On *Y*-axis—share of ETFs in total assets of investment funds [%]. Source: Authors‘ elaboration (data sources described in the Section 3.1).

Figure 3 shows the diffusion patterns of ETFs in the 13 analyzed countries. As may be clearly noticed, there are four countries where the share of the innovative funds reached at least 5% at the end of 2017Q3, implying the most intensive diffusion (starting from the highest share): Switzerland, United Kingdom, Germany and France. In the remaining countries, the share of ETFs was much smaller—their diffusion did not take place or was very limited. Another conclusion which may be drawn from Figure 3 is the increasing inequality in the levels of ETFs diffusion in Europe. In the initial years between-country differences had been significantly smaller than in the final years. Taking into account the assumptions of the diffusion models, it is necessary to evaluate whether the diffusion paths of ETFs in the European countries may be characterized as S-shaped. Unfortunately, the graphical representation in Figure 3 hinders an unequivocal evaluation in the case of most countries. It may be stated, though, that diffusion paths of the German and French markets, and, to lesser extent the Swiss and British, are S-shaped.

Table 5 confirms partially the findings from the graphical analysis with regard to the four most developed ETF markets (it should be emphasized that in all cases estimates are based on the assumption that growth of ETF market follows the S-shaped trajectory). Estimates of diffusion models for France yield misspecifications (substantial overestimates). However, in the case of Germany, Switzerland and the United Kingdom the results can be interpreted (as, for example, values of R^2^ range between 0.78 and 0.985). The highest level of $\kappa_{i}^{ETF}$(upper asymptote) is observed for the UK market, at approximately 28%, which may be regarded as the highest level of diffusion of ETFs, followed by Switzerland with 10% (results for that market are slightly less reliable due to more substantial deviations from the S-shaped trajectory, as indicated by the lower R^2^) and Germany with 7.4%. Estimates of the midpoint, $Tm_{i}^{ETF}$, which may be used to approximately compare the between-country differences in the moment when rapid diffusion started, suggest that, in Switzerland, it occurred somewhat earlier than in Germany (the difference of approximately 3 quarters) and in both countries considerably earlier than in the United Kingdom. Estimated rates of diffusion $\alpha_{i}^{ETF}$ for Switzerland and Germany are also similar and much higher than on the UK market. This means that development of the UK ETF market was relatively slower and started later yet ETFs may be expected to continue their expansion in the upcoming years (the same conclusion may be drawn from the derivative parameter—specific duration $\Delta t_{i}^{ETF}$). Figure 3 shows that UK market is still in the stage of rapid expansion rather than approaching saturation. 

For Italy, the estimated $\kappa_{i}^{ETF}$ is at 0.44 and the intrinsic growth rate (0.16) is comparable to that observed in Germany. Next, for Spain we observe the upper ceiling at 0.59, however with significantly higher—than in Italy, speed of growth as $\alpha_{i}^{ETF}$ = 0.86, which resulted in relatively low value of specific duration, $\Delta t_{i}^{ETF}$ = 5.13 months. For Italy and Spain, the returned R^2^ are 0.84 and 0.45 accordingly. For the remaining nine countries, in cases of seven economies, estimated country-wise diffusion models returned negative parameters (see Table 5), which was mainly caused by unstable ETFs diffusion paths. Negative values of some parameters are reported for Greece, Hungary, Ireland, Norway, Poland, Sweden, and Turkey. Despite the fact that for countries like Sweden or Turkey, the estimated $\kappa_{i}^{ETF}$ parameters seem reliable, both $\alpha_{i}^{ETF}$and $\Delta t_{i}^{ETF}$are negative, which suggests that diffusion of investment funds other than ETFs took place; hence these results shall not be treated as valid. It should be emphasized that the relatively high R^2^ for Italy as well as Germany, Switzerland and United Kingdom suggest that only in these four countries the process of diffusion of ETFs may be approximated by the applied models. This conclusion is further confirmed in the next section.

### 4.3. Tracing ‘Critical Mass’ along ETFs Diffusion Patterns 

With regard to the empirical evidence on the trajectories of ETFs diffusion across selected European economies between 2004 and 2017, presented and discussed in the preceding section, we can draw a general conclusion that the role of ETFs in the aggregate investment funds markets is still rather negligible. Apart from merely a few ‘success stories’ of ETFs diffusion, in the United Kingdom, Switzerland, Germany and France, in case of the remaining countries, the position of ETFs remained negligible. In Greece, Hungary, Ireland, Italy, Norway, Poland, Spain, and Turkey, the average share of ETFs remained below 1%, between 2004Q2 and 2017Q3; while in Sweden it just slightly exceeded 1% (see Table 4). Brief analysis of the country-specific ETFs diffusion patterns and estimated models (see Figure 3 and Table 5), suggests that only in case of the United Kingdom, Switzerland, France and Germany the respective paths were relatively stable over examined time period. We may also notice relative stability of the time-path of the share of ETFs in case of Italy but still the role of this type of financial innovation in the Italian financial system was immaterial—the highest reported share of ETFs was 0.64% in 2017Q3. Visual examination of the trajectories for the other countries confirms high in-time instability; for instance, in case of Hungary, Ireland, or Sweden, ETFs diffusion patterns were marked by random ups and downs. 

In what follows, we aim to uncover whether the critical mass was reached along respective ETFs diffusion patterns across examined markets, which would augment further the logistic growth of their share in total assets of investment funds. With this aim, using the methodological approach discussed in Section 3.2, for each individual ETFs market, we define what follows:

(8) $$N_{i,t}=ETFs_{i,t},$$

where $ETFs_{i,y}$ stands for share of ETFs in total assets of investment funds in the i-th country and the y-th time-period, and:

(9) $$\Phi_{i,t}=\Phi_{i,t}^{ETFs},$$

(10) $$\Omega_{i,t}=\Omega_{i,t}^{ETFs},$$

where $\Phi_{i,t}^{ETFs}$ represents ETFs share replication coefficient for the i-th country and the y-th time-period while $\Omega_{i,t}^{ETFs}$ is the ETFs share marginal growth for the i-th country and the y-th time-period. 

The value of $\Omega_{i,t}^{ETFs}$ for each consecutive time period shows absolute changes in the share of ETFs in total assets of investment funds in the i-th country. The value of $\Phi_{i,t}^{ETFs}$ for each consecutive time period approximates the ETFs share dynamics and thus shows the pace at which ETFs are expanding on the investment funds market.

**Figure 4:**
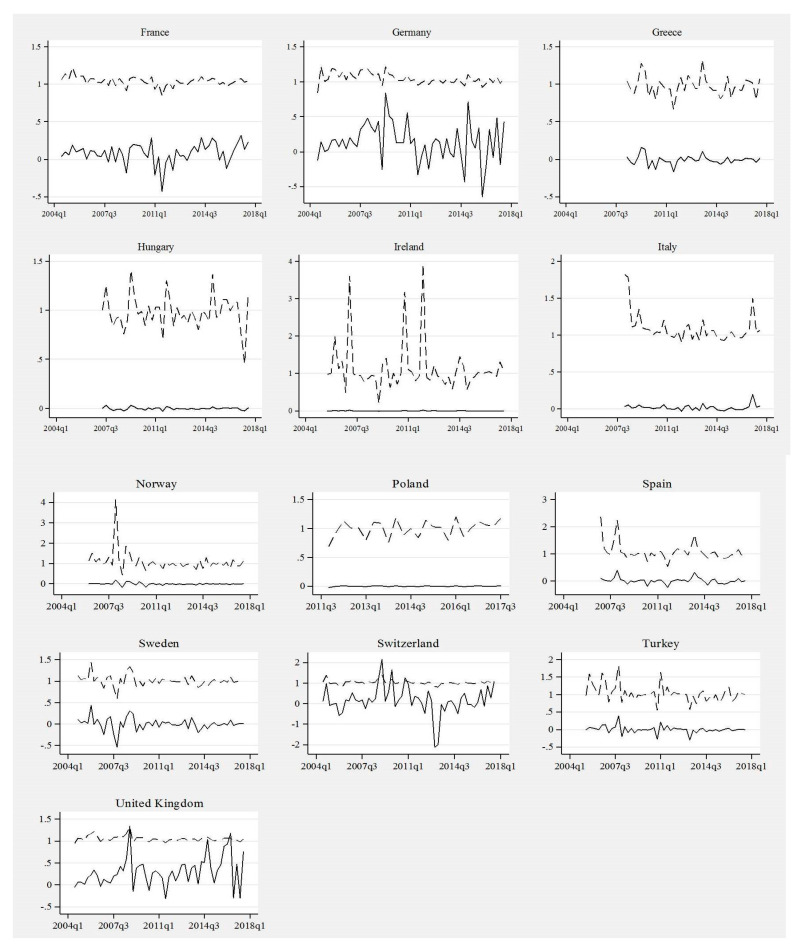
Time patterns of ETFs share marginal growth and replication coefficients. Note: solid line—ETFs share marginal growth ($\Omega_{i,t}^{ETFs}$); dash-line—ETFs share replication coefficient ($\Phi_{i,t}^{ETFs}$). Source: Authors‘ elaboration.

With this methodological framework, we calculate country-wise $\Phi_{i,t}^{ETFs}\;and\;\Omega_{i,t}^{ETFs}$ for each quarter between 2004Q2 and 2017Q3, after which we draw respective $\Phi_{i,t}^{ETFs}\;and\;\Omega_{i,t}^{ETFs}$ time-patterns. As a result, we can identify both numerically and graphically whether the ‘ETFs critical mass’ was reached or not in each individual case. The results of the numerical analysis are summarized in Table A1 in Appendix A and country-specific $\Phi_{i,t}^{ETFs}$ and $\Omega_{i,t}^{ETFs}$ are displayed on Figure 4. 

Simultaneous analysis of Table A1 in Appendix A and country-wise evidence displayed on Figure 4 can be used for identification of those countries where the ETF critical mass was observed between 2004Q1 and 2017Q3. The first thing to note is that the calculated values of $\Phi_{i,t}^{ETFs}$ and $\Omega_{i,t}^{ETFs}$ do not differ significantly across countries. A brief analysis of respective charts in Figure 4 demonstrates that the value of ETFs share marginal growths ($\Omega_{i,t}^{ETFs}$) in particular countries and time periods oscillates between −0.5 and +0.5, and only in a few cases it exceeded 1 (compare charts for Germany, United Kingdom and Switzerland). The latter shows that quarter-by-quarter absolute increases in ETFs share were not significantly high.

Another striking observation is that in Hungary, Ireland, Italy, Norway, Poland, and Spain, the country-wise $\Omega_{i,t}^{ETFs}$ time patterns are flat, showing negligible growths in ETFs share during the examined period. In countries like France, Greece, Sweden, and Turkey, the $\Omega_{i,t}^{ETFs}$ patterns unveil more in-time variability. Finally, in Germany, Switzerland and United Kingdom we observe frequent variations in the values of ETFs share marginal growths. For the last three considered economies, the $\Omega_{i,t}^{ETFs}$ patterns demonstrate relative instability over time suggesting rapid and frequent changes on those markets. Turning to an examination of the ETFs share replication coefficients $\Phi_{i,t}^{ETFs}$, another interesting observation arises. Still, except for a few cases, the value of ETFs share replication coefficients is relatively low (compare data in Table A1 in Appendix A), which inevitably leads to a general conclusion that quarter-by-quarter ETFs share dynamics is rather weak. Apart from just some isolated time periods in Ireland, Norway, Spain and Switzerland, when $\Phi_{i,t}^{ETFs}$ values were slightly higher than 2, in all remaining cases $\Phi_{i,t}^{ETFs}$ lower than 2 is reported showing relatively weak dynamics of ETFs share changes. For countries like Ireland and Hungary, we observe extremely high instability of $\Phi_{i,t}^{ETFs}$ paths, which shows abruptly and radically changing dynamics of ETFs share growth. Conversely, in countries like France, Germany, Switzerland and United Kingdom the $\Phi_{i,t}^{ETFs}$ trajectories unveil relative in-time stability, which reflects stable growth of ETFs share on these markets. 

Analysis of graphical and numerical results obtained in this section shows whether ETF critical mass was reached in the examined economies. The ETF critical mass can be identified if $\Phi_{i,t}^{ETFs}$ and $\Omega_{i,t}^{ETFs}$ patterns converge and finally intersect, in the time period during which $\Phi_{i,t}^{ETFs}$ = $\Omega_{i,t}^{ETFs}$ is observed. Such evidence would show that initially the values of ETFs share replication coefficients are higher than ETFs share marginal growth; however, over time the former coefficient decreases while the latter increases. It would mean that the gap between $\Phi_{i,t}^{ETFs}$ and $\Omega_{i,t}^{ETFs}$ is being gradually eradicated until the $\Phi_{i,t}^{ETFs}$ = $\Omega_{i,t}^{ETFs}$, and during consecutive time periods we would observe $\Phi_{i,t}^{ETFs}$ < $\Omega_{i,t}^{ETFs}$. Our graphical and numerical evidence demonstrates that this is not the case in any of considered economies. The $\Phi_{i,t}^{ETFs}$ and $\Omega_{i,t}^{ETFs}$ paths have intersected only in case of Switzerland and United Kingdom, but even in these two cases, claiming that the ETF critical mass was achieved would be an overstatement. Claiming that the ETF critical mass was reached in these countries would imply that $\Phi_{i,t}^{ETFs}$ and $\Omega_{i,t}^{ETFs}$ patterns gradually diverge after reaching the critical mass, and clearly that is not the case. In all remaining countries the $\Phi_{i,t}^{ETFs}$ and $\Omega_{i,t}^{ETFs}$ patterns did not intersect; the gap between values of $\Phi_{i,t}^{ETFs}$ and $\Omega_{i,t}^{ETFs}$ is persistent suggesting that all these economies are locked in the low-level trap regarding the ETFs diffusion and their role in the investment funds industry. The latter also shows that, in a great majority of examined countries, increases of the share of ETFs in total assets of investment funds are insubstantial and this type of financial products did not gain substantial popularity. 

Conclusions drawn from the analysis of the $\Phi_{i,t}^{ETFs}$ and ${}_{i,t}^{ETFs}$ patterns, conducted in order to determine whether the ETF critical mass was reached in the selected countries, may also be discussed with regard to the potential role of ETFs in the financial systems. With the exception of Switzerland and the United Kingdom, where the possibility of achieving the critical mass by ETFs should not be disregarded (yet it has still been not observed), in the remaining countries innovative funds have not reached the level at which their further logistic growth and self-perpetuating diffusion would be possible. Results obtained using the diffusion and critical mass framework may be further put in the context of the between-country differences in the size of the local capital markets. Due to the attributes of the most European ETFs (which are usually passive equity funds) the key segment to be considered is the stock markets. According to the World Bank’s Global Financial Development Database over 2004–2016, the average stock market capitalization in relation to GDP exceeded 100% in the United Kingdom and 200% in Switzerland. The total value of the annual turnover on the stock market in relation to GDP was on average over 90% in the United Kingdom and close to 120% in Switzerland. These values can be compared with the corresponding indicators for the remaining countries. For example, in case of Germany the average values of both measures were between 40 and 50% of GDP; in case of France, they were higher (between 60 and 75% of GDP) but still much lower than for the two aforementioned countries. The discrepancies become even more substantial when we take into account the countries with the least developed ETF markets, in which the stock markets are much smaller in all dimensions (in particular in terms of turnover). The results of this brief analysis show that the role of ETFs is positively linked to the importance of the stock market in relation to the size of the entire economy; in other words, the underdevelopment of the local equity market may hinder the expansion of ETFs and their potential to reach the critical mass. These conclusions can be explained by referring to the fundamental attributes of the leading types of European ETFs, i.e., passive equity ETFs, which in their creation/redemption mechanisms, as well as potential benefits for investors, are strongly linked to the activities on the markets for the underlying assets—equities. 

As a result, we may state that it is highly improbable for ETFs to have significantly impacted (either positively or negatively) the financial systems of European countries or to exert such influence in the upcoming few years. Effects of the ETFs diffusion, presented in the Section 2.2, therefore seem limited to the largest ETF markets such as the United States. This does not mean that ETFs have not affected the financial systems in any way, yet such impacts have been limited by their low share in the total assets of investment funds and their high variability. Moreover, it should be underlined that the speed of ETFs diffusion in the future may change (potentially increase) due to changes in their market environment such as new regulatory developments. Finally, it must be emphasized that a determination of the exact linkages and their intensity lies outside the scope of this study.

## 5. Conclusions

This paper was designed to contribute to the present state of knowledge on the process of ETFs diffusion across the European financial markets. With this aim, we have selected 13 financial markets, for which balanced time series on the total assets of both ETFs and investment funds were available. Our analysis spans between 2004 and 2017, and within this time period we examine quarterly data on total assets of investment funds, total assets of ETFs, and share of ETFs in total assets of investment funds. The main focus of our research concentrates on the last variable. Our major target was two-fold. First, we aimed to develop country-wise ETFs diffusion patters, and to complete the latter we applied innovation diffusion models. Such an approach, still rare in this area of research, was used to unveil specific features of the ETFs diffusion paths and to demonstrate in-time dynamics of this process. Second, we used a novel methodological approach to calculate the ‘critical mass’ that provided more profound insight into the dynamic of the process of ETFs diffusion. Examination of the critical mass along ETFs diffusion trajectories for each individual country made it possible to determine the time and ETFs share in total assets of investment funds that enhanced the process of logistic growth of ETFs. 

Our research leads to several conclusions. First, we have shown that, during analyzed period, in 10 economies the total growth of the share of ETFs in assets of investment funds was observed, while the highest dynamics of the process was reported in Italy, United Kingdom and Germany. Only in Greece, Hungary, and Poland have we observed drops in ETFs share. The highest ETFs share in assets of investment funds was demonstrated for the United Kingdom, Switzerland, Germany, and France, with some significant between-country differences. In the remaining nine countries (except Sweden), the maximum observed ETFs shares did not exceed 1%, suggesting that in these financial markets the role of ETFs was still negligible between 2004 and 2017. A closer look at the estimates of diffusion models shows that, only for Germany, Italy, Spain, Switzerland, and the United Kingdom, the returned parameters are conclusive. Estimated parameters show that the highest intrinsic growth rates are reported for Spain and Switzerland, with the highest ceiling (upper asymptote) for the United Kingdom and Switzerland, suggesting that in these two countries the process of growing share of ETFs in assets of investment funds is most dynamic and follows relatively stable development path. Unreliable estimates of the diffusion models in the remaining cases are due to two main reasons, namely an indecently low share of ETFs and/or very unstable diffusion paths that are marked by sudden ups and downs; both heavily violate model estimates. As for the critical mass identification, results of the analysis imply that the achievement of the critical mass can convincingly be predicted exclusively for Switzerland and United Kingdom. These results coincide with the diffusion models estimates, and demonstrate that only in these two cases the process of increasing share of ETFs in assets of investment funds is stable enough so that entering logistic growth phase along development paths is possible.

The results of our study show that, despite the substantial growth of both assets of ETFs and their share in the aggregate market for the investment funds in most of the analyzed countries, their role in the financial systems (and as a consequence also in the local economies) remains immaterial. Moreover, even in the case of the two countries for which reaching the critical mass level of market share of ETFs seems plausible (assuming the continuation of the current diffusion trend), it would be an overstatement to conclude that they may significantly influence the local financial systems, either currently or in the near future. Further research could address the issue of the factors that boost or limit the diffusion of ETFs, in particular in the context of reaching the critical mass, and explanation of the between-country differences in the discussed processes. Furthermore, the results of our analysis have a number of potentially important implications that can be identified both at the level of investors and the entire economy. The still limited development of the ETF markets in most analyzed European countries means that investors have limited access to financial products that in many aspects are usually more beneficial than the conventional investment funds (e.g., in terms of transparency or liquidity) and can be used to reach various aims due to their broad range of applications. In terms of the local economies, the identified problems of the ETF markets to reach the critical mass levels mean that the systemic benefits of ETFs remain to be gained, including growth of the accessibility of the financial markets (with possible far-reaching social and economic consequences) and increased financial openness through the cross-listing of ETFs and activities of ETFs with non-domestic holdings which may contribute positively to, e.g., diversification opportunities of investors or capital-raising possibilities for the enterprises.

## Figures and Tables

**Table 1:** Main differences between ETFs and mutual funds. Authors’ compilation based on [1,4,12,13,15,19,20,21,22,23].

Feature	ETFs	Mutual Funds
Market structure.	Two segments: primary and secondary market. Secondary market operations can be conducted on stock exchanges or similar trading venues during their trading hours; transactions include short selling and other available for listed securities.	No separation (transactions conducted directly between holders of the units and funds). There are various channels of distribution but units are unavailable on stock exchanges—lower liquidity compared to ETFs.
Valuation (price) of fund’s units.	Dual:	Single: net asset value (NAV) calculated by the fund; less frequent valuation than in case of ETFs.
	1. primary market: net asset value (NAV) calculated by the fund; 2. secondary market: price influenced by the interaction of demand and supply as well as actions of authorized participants—intraday continuous pricing.	
Transparency of the fund’s portfolio.	Portfolio’s composition typically published daily.	Portfolio’s composition typically published monthly or quarterly.
Derivatives based on the fund’s units.	Available	Unavailable
Accessibility.	ETFs may be accessed by buying a preferred number of units (one or more), with the desired exposure, for instance, to the foreign assets.	Limits of minimum investment may be imposed. Investing into foreign mutual funds is more complicated than in case of foreign-listed ETFs (due to, e.g., cross-listing of ETFs).

**Table 2:** Selected concepts of ETFs as financial innovations in the literature. Direct quotes in italics. Authors’ elaboration.

Deville [19]	ETFs as Highly Successful Financial Innovations
Gastineau [21]	ETFs as a milestone in the human history.
Agapova [20]	Discussion of the innovative features of ETFs.
Schoenfeld [24]	ETFs as *innovative financial vehicles*, their launch *revolutionary*.
Hill [25]	ETFs as *product innovation*.
Madhavan [13]	ETFs as an example of authentic financial innovations.
Amenc, Goltz and Le Sourd [26]	ETFs as one of the *greatest financial innovations of recent years.*
Ben-David, Franzoni and Moussawi [22]	ETFs as financial innovations, new types of ETFs as *innovations within innovations*.

**Table 3:** Selected possible negative effects of the diffusion of ETFs in the financial system. Authors’ compilation based on [10,13,22,34,35,36,37,38,39,40,41,42,43].

Negative Effects (Risks)
Shock propagation (transmission) from the ETF markets to the markets for the underlying assets; it applies to the non-fundamental shocks, for instance caused by the fluctuating demand for the shares of ETFs. Flash Crash is sometimes presented as an example.
Cross-listing of ETFs can lead to transmission of shocks between markets for ETFs and assets located in various countries that are tracked by the funds or held in their portfolios.
Commodity ETFs may magnify speculation on the commodity markets, increasing their volatility.
Risks associated with synthetic ETFs:
1. counterparty risk: default of the swap contract’s counterparty or its failure to deliver the agreed returns; 2. collateral risk: income from the sale of the collateral or returns generated by the collateral assets too low to cover the necessary costs (in case of the swap’s counterparty’s default); 3. insufficient transparency and disclosure concerning the swap transactions.
A special category of synthetic ETFs, i.e., geared ETFs (with leveraged, inverse or leveraged-inverse returns) may generate additional risks: 1. increased volatility of the associated markets (e.g., equity markets in case of equity geared funds); 2. augmented short-term speculation and exaggerated sensitivity of the ETF’s shares to company-specific information.

**Table 4:** Assets of investment funds and ETFs—summary statistics. Period 2004–2017, quarterly data. Authors‘ calculations (data sources described in the Section 3.1). Note: for absolute growth and total growth rate calculations—base period is the first period when considered value differs from zero; average quarterly dynamic is calculated as geometric mean.

Country	# of Obs.	Mean	Maximum	Minimum	Average Quarterly Dynamic	Absolute Change	Total Growth Rate [%]
total assets of investment funds [mln USD]							
France	54	1,909,097	2,283,095	1,322,281	101.0	927,690.1	70.2
Germany	54	1,589,481	2,347,751	1,019,214	101.6	1,328,537	130.3
Greece	54	18,228	48,452	7169	97.2	−30,252.8	−77.3
Hungary	54	15,782	23,090	4100	103.3	18,586.41	453.3
Ireland	54	1,411,250	2,676,116	503,020	103.2	2,173,096	432.0
Italy	54	368,166	537,204	234,015	99.5	−105,976	−22.4
Norway	54	80,667	138,036	23,416	103.4	114,620.5	489.5
Poland	54	42,896	76,281	9389	104.0	64,673.7	688.8
Spain	54	289,251	411,437	190,378	100.4	68,572.3	25.6
Sweden	54	218,359	387,307	94,084	102.7	293,223.5	311.7
Switzerland	54	347,856	657,544	100,905	103.5	530,112.5	525.4
Turkey	54	25,749	33,850	16,809	101.2	14,344.06	84.4
United Kingdom	54	1,161,377	1,850,704	546,704	102.3	1,304,000	238.5
total assets of ETFs [mln USD]							
France	54	49,516	111,308	8637	104.9	102,634.7	1183.3
Germany	54	86,253	188,704	7170	106.1	180,708.4	2259.9
Greece	54	37.8	197.4	0.0	93.6	−181.4	−91.9
Hungary	54	11.03	27.5	0.0	97.9	−8.9	−59.5
Ireland	54	125.8	32	0.0	103.5	136.2	430.6
Italy	54	757	2344	0.0	120.1	2343.3	220,048.3
Norway	54	134	342	0.0	105.9	126.9	1392.2
Poland	54	16.2	92.7	0.0	96.7	−50.0	−60.9
Spain	54	1268	2892	0.0	118.3	1820.73	225,571.8
Sweden	54	2364	3471	804	102.9	2667.06	331.5
Switzerland	54	30,738	73,298	2447	106.6	70,851.0	2894.3
Turkey	54	97.9	229	0.0	102.1	31.0	176.2
United Kingdom	54	100,932	312,100	6028	107.7	306,071.3	5076.8
share of ETFs in total assets of investment funds [%]							
France	54	2.53	4.94	0.65	103.9	4.3	654.2
Germany	54	4.98	8.03	0.66	104.5	7.3	924.5
Greece	54	0.29	0.83	0.0	96.8	−0.4	−70.9
Hungary	54	0.064	0.15	0.0	96.5	−0.1	−77.7
Ireland	54	0.008	0.025	0.0	100.4	0.001	23.2
Italy	54	0.24	0.64	0.0	121.0	0.6	1657.0
Norway	54	0.16	0.47	0.0	103.2	0.1	333.7
Poland	54	0.03	0.22	0.0	94.3	−0.2	−80.4
Spain	54	0.45	0.99	0.0	118.3	0.5	664.2
Sweden	54	1.08	1.55	0.0	100.2	0.1	11.0
Switzerland	54	7.69	13.18	2.39	103.0	9.2	378.8
Turkey	54	0.38	0.86	0.0	101.1	0.1	72.3
United Kingdom	54	7.39	16.8	1.04	105.3	15.8	1429.3

**Table 5:** Estimates of country-level models of ETFs diffusion. Period 2004–2017, quarterly data. Note: in italics—misspecifications; due to breaks in time series, in some countries period of analysis is shorter. Authors’ estimates.

	France	Germany	Greece	Hungary	Ireland	Italy	Norway	Poland	Spain	Sweden	Switzerland	Turkey	United Kingdom
$\kappa_{i}^{ETF}$ **(ceiling/upper asymptote)**	*35 565.9 [overestimates]*	7.36	*1.05*	*0.08*	*0.011*	0.44	*0.21*	*102 488.1*	0.59	*1.2*	10.5	*0.49*	27.8
$Tm_{i}^{ETF}\;(\beta_{i}^{ETF}$) **(midpoint)**	*395 [overestimates]*	17.7	*26.3*	*137.6*	*48.73*	23.5	*48.68*	*−49.24*	13.63	*58.4*	15.3	*44.7*	47.3
$\alpha_{i}^{ETF}$ **(rate of diffusion)**	*0.026*	0.173	*−0.06*	*−0.63*	*−0.19*	0.16	*−0.18*	*−0.17*	0.86	*−0.13*	0.191	*−0.28*	0.061
$\Delta t_{i}^{ETF}$ **(specific duration)**	*168 [overestimates]*	25.4	*−73.08*	*−6.89*	*−22.3*	26.5	*−23.32*	*−25.5*	5.13	*−34.8*	23	*−15.9*	72.1
**R2 of the model**	*0.97*	0.985	*0.76*	*0.18*	*0.09*	0.84	*0.12*	*0.11*	0.45	*0.27*	0.78	*0.21*	0.98
**# of obs.**	*54*	54	*39*	*43*	*51*	43	*48*	*29*	47	*53*	54	*51*	54

## Appendix A

Table A1 ETFs share marginal growth $\Omega_{i,y}^{\mathrm{ETFs}}$ and replication coefficients $\Phi_{i,y}^{\mathrm{ETFs}}$ country-wise calculations. Period 2004–2017, quarterly data.

**Table:** (**a**)

Time	France			Germany			Greece			Huingary			Ireland			Italy			Norway		
	$ETFs_{i,y}$	$\Omega_{i.y}^{ETFs}$	$\Phi_{i.y}^{ETFs}$	$ETFs_{i,y}$	$\Omega_{i.y}^{ETFs}$	$\Phi_{i.y}^{ETFs}$	$ETFs_{i,y}$	$\Omega_{i.y}^{ETFs}$	$\Phi_{i.y}^{ETFs}$	$ETFs_{i,y}$	$\Omega_{i.y}^{ETFs}$	$\Phi_{i.y}^{ETFs}$	$ETFs_{i,y}$	$\Omega_{i.y}^{ETFs}$	$\Phi_{i.y}^{ETFs}$	$ETFs_{i,y}$	$\Omega_{i.y}^{ETFs}$	$\Phi_{i.y}^{ETFs}$	$ETFs_{i,y}$	$\Omega_{i.y}^{ETFs}$	$\Phi_{i.y}^{ETFs}$
2004:2	0.66	-	-	0.78	-	-	0.00	-	-	0.00	-	-	0.00	-	-	0.00	-	-	0.00	-	-
2004:3	0.70	0.04	1.06	0.66	−0.12	0.84	0.00	-	-	0.00	-	-	0.00	-	-	0.00	-	-	0.00	-	-
2004:4	0.79	0.10	1.14	0.81	0.14	1.22	0.00	-	-	0.00	-	-	0.00	-	-	0.00	-	-	0.00	-	-
2005:1	0.85	0.06	1.07	0.81	0.01	1.01	0.00	-	-	0.00	-	-	0.00	-	-	0.00	-	-	0.00	-	-
2005:2	1.04	0.19	1.22	0.84	0.03	1.04	0.00	-	-	0.00	-	--	0.01	-	-	0.00	-	-	0.00	-	-
2005:3	1.14	0.10	1.09	1.01	0.16	1.19	0.00	-	-	0.00	-	-	0.00	0.00	0.97	0.00	-	-	0.00	-	-
2005:4	1.26	0.12	1.10	1.18	0.18	1.18	0.00	-	-	0.00	-	-	0.00	0.00	1.00	0.00	-	-	0.02	-	-
2006:1	1.40	0.14	1.11	1.25	0.07	1.06	0.00	-	-	0.00	-	-	0.01	0.00	1.99	0.00	-	-	0.03	0.00	1.13
2006:2	1.40	0.00	1.00	1.44	0.18	1.15	0.00	-	-	0.00	-	-	0.01	0.00	1.12	0.00	-	-	0.04	0.01	1.52
2006:3	1.52	0.11	1.08	1.47	0.04	1.03	0.00	-	-	0.00	-	-	0.01	0.00	1.33	0.00	-	-	0.04	0.00	1.07
2006:4	1.63	0.11	1.07	1.67	0.20	1.14	0.00	-	-	0.00	-	-	0.01	−0.01	0.47	0.00	-	-	0.05	0.01	1.25
2007:1	1.67	0.04	1.03	1.80	0.12	1.07	0.00	-	-	0.12	-	-	0.03	0.02	3.62	0.00	-	-	0.05	0.00	0.97
2007:2	1.71	0.04	1.02	1.87	0.07	1.04	0.00	-	-	0.12	0.00	1.00	0.03	0.00	1.00	0.00	-	-	0.05	0.00	1.03
2007:3	1.82	0.12	1.07	2.19	0.32	1.17	0.00	-	-	0.15	0.03	1.25	0.02	0.00	0.92	0.00	-	-	0.07	0.02	1.33
2007:4	1.79	−0.04	0.98	2.58	0.39	1.18	0.00	-	-	0.15	0.00	0.99	0.02	0.00	0.95	0.04	-	-	0.06	−0.01	0.92
2008:1	1.95	0.17	1.09	3.06	0.48	1.19	0.62	-	-	0.13	−0.02	0.85	0.02	−0.01	0.77	0.07	0.03	1.82	0.26	0.20	4.13
2008:2	1.92	−0.04	0.98	3.41	0.36	1.12	0.65	0.03	1.04	0.12	−0.01	0.92	0.01	0.00	0.86	0.12	0.05	1.79	0.29	0.03	1.12
2008:3	2.06	0.15	1.08	3.69	0.28	1.08	0.60	−0.04	0.93	0.11	−0.01	0.93	0.01	0.00	0.95	0.13	0.01	1.11	0.13	−0.17	0.43
2008:4	2.12	0.06	1.03	4.13	0.44	1.12	0.53	−0.07	0.88	0.08	−0.03	0.76	0.01	0.00	0.92	0.15	0.02	1.13	0.24	0.11	1.87
2009:1	1.94	−0.18	0.91	3.88	−0.25	0.94	0.55	0.02	1.04	0.07	−0.01	0.90	0.00	−0.01	0.21	0.20	0.05	1.35	0.37	0.13	1.57
2009:2	2.09	0.15	1.08	4.72	0.84	1.22	0.70	0.16	1.28	0.10	0.03	1.40	0.00	0.00	1.25	0.22	0.02	1.10	0.39	0.02	1.05
2009:3	2.29	0.20	1.09	5.22	0.50	1.11	0.84	0.13	1.19	0.12	0.02	1.15	0.00	0.00	1.41	0.23	0.02	1.08	0.34	−0.05	0.88
2009:4	2.47	0.19	1.08	5.68	0.46	1.09	0.71	−0.13	0.85	0.11	0.00	0.96	0.00	0.00	0.62	0.25	0.02	1.07	0.45	0.11	1.31
2010:1	2.65	0.17	1.07	5.80	0.12	1.02	0.69	−0.02	0.98	0.11	0.00	0.99	0.00	0.00	1.01	0.25	0.00	1.01	0.47	0.02	1.04
2010:2	2.73	0.08	1.03	5.93	0.12	1.02	0.55	−0.14	0.80	0.10	−0.02	0.85	0.00	0.00	0.71	0.26	0.01	1.04	0.31	−0.16	0.65
2010:3	2.76	0.02	1.01	6.06	0.13	1.02	0.58	0.02	1.04	0.10	0.00	1.05	0.00	0.00	1.00	0.27	0.01	1.03	0.30	−0.01	0.98
2010:4	3.04	0.28	1.10	6.61	0.55	1.09	0.56	−0.01	0.98	0.09	-0.01	0.90	0.01	0.00	3.18	0.33	0.06	1.21	0.33	0.03	1.10
2011:1	2.83	−0.21	0.93	6.72	0.11	1.02	0.52	−0.04	0.93	0.09	0.00	1.03	0.01	0.00	1.10	0.33	0.00	1.00	0.30	−0.02	0.93
2011:2	2.86	0.03	1.01	6.91	0.19	1.03	0.49	−0.03	0.94	0.10	0.00	1.03	0.01	0.00	1.04	0.32	−0.01	0.98	0.30	0.00	1.00
2011:3	2.43	−0.43	0.85	6.58	−0.33	0.95	0.33	−0.17	0.66	0.07	−0.03	0.70	0.01	0.00	0.80	0.31	−0.01	0.96	0.23	−0.08	0.75
2011:4	2.38	−0.05	0.98	6.51	−0.07	0.99	0.30	−0.02	0.93	0.09	0.02	1.30	0.01	0.00	0.93	0.33	0.02	1.05	0.25	0.02	1.10
2012:1	2.43	0.05	1.02	6.62	0.10	1.02	0.33	0.03	1.09	0.10	0.01	1.11	0.02	0.02	3.93	0.29	−0.03	0.90	0.23	−0.02	0.92
2012:2	2.28	−0.15	0.94	6.37	−0.25	0.96	0.30	−0.03	0.91	0.08	−0.02	0.84	0.02	0.00	0.89	0.32	0.03	1.10	0.24	0.00	1.02
2012:3	2.42	0.13	1.06	6.48	0.11	1.02	0.34	0.04	1.12	0.08	0.00	1.03	0.02	0.00	0.82	0.37	0.05	1.14	0.21	−0.02	0.90
2012:4	2.46	0.04	1.02	6.66	0.18	1.03	0.35	0.02	1.04	0.08	−0.01	0.91	0.02	0.00	1.21	0.35	−0.02	0.94	0.22	0.01	1.05
2013:1	2.51	0.05	1.02	6.80	0.14	1.02	0.33	−0.02	0.94	0.07	0.00	0.95	0.02	0.00	0.93	0.36	0.01	1.04	0.19	−0.03	0.84
2013:2	2.49	−0.02	0.99	6.70	−0.10	0.99	0.31	−0.02	0.94	0.06	−0.01	0.87	0.02	0.00	0.88	0.34	−0.02	0.93	0.18	−0.01	0.96
2013:3	2.59	0.10	1.04	6.89	0.19	1.03	0.42	0.10	1.32	0.06	0.00	1.00	0.01	0.00	0.70	0.41	0.07	1.21	0.17	−0.01	0.97
2013:4	2.76	0.17	1.07	6.88	−0.01	1.00	0.43	0.02	1.04	0.06	−0.01	0.92	0.01	0.00	0.91	0.40	−0.01	0.99	0.16	−0.01	0.93
2014:1	2.86	0.10	1.04	6.80	−0.08	0.99	0.42	−0.02	0.96	0.05	−0.01	0.80	0.01	0.00	0.58	0.43	0.02	1.06	0.12	−0.05	0.72
2014:2	3.15	0.29	1.10	7.13	0.33	1.05	0.38	−0.03	0.92	0.05	0.00	1.00	0.01	0.00	1.04	0.45	0.03	1.06	0.14	0.02	1.18
2014:3	3.28	0.13	1.04	7.10	−0.03	1.00	0.35	−0.03	0.92	0.04	0.00	0.96	0.01	0.00	1.45	0.44	−0.01	0.97	0.10	−0.03	0.76
2014:4	3.45	0.17	1.05	6.67	−0.43	0.94	0.29	−0.07	0.81	0.04	−0.01	0.89	0.01	0.00	1.20	0.41	−0.03	0.94	0.14	0.03	1.30
2015:1	3.74	0.28	1.08	7.39	0.72	1.11	0.25	−0.04	0.87	0.05	0.01	1.37	0.01	0.00	0.56	0.38	−0.03	0.93	0.11	−0.02	0.84
2015:2	3.97	0.23	1.06	7.54	0.16	1.02	0.28	0.03	1.11	0.05	0.00	0.93	0.00	0.00	0.83	0.38	−0.01	0.98	0.12	0.00	1.03
2015:3	3.96	−0.01	1.00	7.59	0.05	1.01	0.23	−0.05	0.82	0.05	0.00	0.95	0.00	0.00	0.91	0.39	0.02	1.05	0.11	−0.01	0.95
2015:4	4.06	0.11	1.03	7.93	0.34	1.05	0.22	−0.01	0.97	0.05	0.01	1.11	0.00	0.00	1.03	0.38	−0.01	0.97	0.12	0.01	1.11
2016:1	3.94	−0.12	0.97	7.29	−0.64	0.92	0.20	−0.01	0.93	0.06	0.01	1.11	0.00	0.00	1.00	0.37	−0.02	0.96	0.11	−0.01	0.91
2016:2	3.94	0.00	1.00	7.08	−0.22	0.97	0.19	−0.02	0.92	0.06	0.00	0.99	0.00	0.00	1.02	0.35	−0.01	0.96	0.12	0.01	1.07
2016:3	4.05	0.11	1.03	7.39	0.32	1.04	0.20	0.01	1.05	0.06	0.00	1.07	0.00	0.00	1.04	0.36	0.01	1.02	0.09	−0.03	0.76
2016:4	4.27	0.21	1.05	7.31	−0.08	0.99	0.21	0.01	1.04	0.07	0.01	1.09	0.00	0.00	1.00	0.39	0.03	1.07	0.11	0.02	1.21
2017:1	4.59	0.32	1.07	7.80	0.49	1.07	0.21	0.00	1.02	0.05	−0.02	0.74	0.00	0.00	0.92	0.58	0.19	1.50	0.10	−0.01	0.90
2017:2	4.72	0.13	1.03	7.61	−0.18	0.98	0.17	−0.04	0.80	0.02	−0.03	0.45	0.01	0.00	1.31	0.60	0.02	1.03	0.09	−0.01	0.89
2017:3	4.95	0.23	1.05	8.04	0.42	1.06	0.18	0.01	1.08	0.03	0.00	1.16	0.01	0.00	1.06	0.64	0.04	1.06	0.10	0.01	1.12

**Table:** (**b**)

	Poland			Spain			Sweden			Switzerland			Turkey			United Kingdom		
Time	$ETFs_{i,y}$	$\Omega_{i.y}^{ETFs}$	$\Phi_{i.y}^{ETFs}$	$ETFs_{i,y}$	$\Omega_{i.y}^{ETFs}$	$\Phi_{i.y}^{ETFs}$	$ETFs_{i,y}$	$\Omega_{i.y}^{ETFs}$	$\Phi_{i.y}^{ETFs}$	$ETFs_{i,y}$	$\Omega_{i.y}^{ETFs}$	$\Phi_{i.y}^{ETFs}$	$ETFs_{i,y}$	$\Omega_{i.y}^{ETFs}$	$\Phi_{i.y}^{ETFs}$	$ETFs_{i,y}$	$\Omega_{i.y}^{ETFs}$	$\Phi_{i.y}^{ETFs}$
2004:2	0.00	-	-	0.00	-	-	0.00	-	-	2.43	-	-	0.00	-	-	1.10	-	-
2004:3	0.00	-	-	0.00	-	-	0.81	-	-	2.55	0.12	1.05	0.00	-	-	1.05	−0.05	0.95
2004:4	0.00	-	-	0.00	-	-	0.91	0.10	1.12	3.52	0.98	1.38	0.00	-	-	1.11	0.06	1.06
2005:1	0.00	-	-	0.00	-	-	0.93	0.02	1.02	3.43	−0.10	0.97	0.09	-	-	1.17	0.06	1.05
2005:2	0.00	-	-	0.00	-	-	0.99	0.06	1.07	3.40	−0.02	0.99	0.09	0.00	0.98	1.18	0.01	1.01
2005:3	0.00	-	-	0.00	-	-	1.01	0.02	1.02	3.42	0.01	1.00	0.14	0.05	1.60	1.35	0.16	1.14
2005:4	0.00	-	-	0.00	-	-	1.44	0.44	1.43	2.84	−0.57	0.83	0.18	0.04	1.30	1.58	0.23	1.17
2006:1	0.00	-	-	0.00	-	-	1.43	−0.02	0.99	2.39	−0.45	0.84	0.20	0.02	1.12	1.92	0.34	1.21
2006:2	0.00	-	-	0.00	-	-	1.54	0.12	1.08	2.58	0.19	1.08	0.20	0.00	0.99	2.14	0.22	1.11
2006:3	0.00	-	-	0.07	-	-	1.52	−0.02	0.99	2.72	0.14	1.06	0.33	0.13	1.62	2.11	−0.03	0.99
2006:4	0.00	-	-	0.17	0.10	2.38	1.27	−0.25	0.84	3.25	0.53	1.19	0.47	0.14	1.43	2.24	0.13	1.06
2007:1	0.00	-	-	0.21	0.04	1.22	1.38	0.11	1.09	3.42	0.17	1.05	0.37	−0.10	0.79	2.31	0.07	1.03
2007:2	0.00	-	-	0.21	0.01	1.04	1.55	0.17	1.12	3.51	0.09	1.03	0.40	0.03	1.08	2.35	0.05	1.02
2007:3	0.00	-	-	0.21	0.00	0.98	1.30	−0.25	0.84	3.69	0.18	1.05	0.47	0.07	1.18	2.56	0.21	1.09
2007:4	0.00	-	-	0.31	0.10	1.50	0.76	−0.54	0.59	3.44	−0.25	0.93	0.86	0.39	1.82	2.80	0.24	1.09
2008:1	0.00	-	-	0.70	0.39	2.25	0.82	0.05	1.07	3.70	0.26	1.08	0.66	−0.20	0.76	3.22	0.43	1.15
2008:2	0.00	-	-	0.75	0.05	1.07	0.73	−0.09	0.89	3.78	0.07	1.02	0.73	0.08	1.12	3.54	0.32	1.10
2008:3	0.00	-	-	0.76	0.01	1.01	0.90	0.17	1.24	4.00	0.22	1.06	0.65	−0.08	0.89	4.13	0.59	1.17
2008:4	0.00	-	-	0.64	−0.12	0.84	1.21	0.30	1.34	5.27	1.27	1.32	0.69	0.04	1.06	5.48	1.34	1.33
2009:1	0.00	-	-	0.64	0.00	1.00	1.44	0.24	1.20	7.43	2.16	1.41	0.59	−0.10	0.86	5.33	−0.15	0.97
2009:2	0.00	-	-	0.61	−0.04	0.94	1.26	−0.19	0.87	7.55	0.12	1.02	0.58	−0.01	0.99	5.72	0.39	1.07
2009:3	0.00	-	-	0.61	0.00	1.01	1.25	−0.01	1.00	8.12	0.57	1.08	0.56	−0.02	0.97	6.16	0.44	1.08
2009:4	0.00	-	-	0.64	0.03	1.05	1.11	−0.14	0.89	9.77	1.65	1.20	0.56	0.00	1.00	6.63	0.47	1.08
2010:1	0.00	-	-	0.68	0.04	1.06	1.13	0.02	1.02	9.61	−0.16	0.98	0.55	−0.01	0.98	6.76	0.13	1.02
2010:2	0.00	-	-	0.49	−0.19	0.72	1.17	0.04	1.04	9.78	0.16	1.02	0.56	0.01	1.02	6.63	-0.13	0.98
2010:3	0.22	-	-	0.52	0.02	1.05	1.13	−0.04	0.96	10.14	0.37	1.04	0.61	0.05	1.09	6.90	0.28	1.04
2010:4	0.22	0.00	1.00	0.47	−0.04	0.92	1.22	0.09	1.08	11.40	1.26	1.12	0.33	−0.28	0.55	7.22	0.32	1.05
2011:1	0.22	−0.01	0.97	0.51	0.04	1.08	1.14	−0.08	0.94	12.33	0.93	1.08	0.55	0.21	1.65	7.47	0.25	1.03
2011:2	0.22	0.00	1.01	0.56	0.05	1.09	1.19	0.05	1.05	12.21	−0.12	0.99	0.53	−0.01	0.98	7.63	0.16	1.02
2011:3	0.06	−0.16	0.27	0.49	−0.06	0.89	1.22	0.03	1.02	12.58	0.36	1.03	0.66	0.12	1.23	7.31	−0.32	0.96
2011:4	0.04	−0.02	0.69	0.26	−0.23	0.53	1.26	0.04	1.04	12.86	0.28	1.02	0.62	-0.03	0.95	7.49	0.17	1.02
2012:1	0.04	0.00	0.94	0.23	−0.03	0.89	1.24	−0.02	0.98	12.89	0.03	1.00	0.67	0.04	1.07	7.80	0.32	1.04
2012:2	0.04	0.01	1.14	0.25	0.02	1.08	1.22	−0.02	0.98	12.41	−0.48	0.96	0.69	0.02	1.03	7.89	0.09	1.01
2012:3	0.04	0.00	1.01	0.30	0.05	1.20	1.19	−0.03	0.97	13.05	0.64	1.05	0.71	0.02	1.03	8.11	0.21	1.03
2012:4	0.04	0.00	1.02	0.32	0.02	1.07	1.19	0.00	1.00	13.18	0.13	1.01	0.70	−0.01	0.99	8.56	0.45	1.06
2013:1	0.04	−0.01	0.80	0.36	0.04	1.11	1.29	0.10	1.08	11.06	−2.12	0.84	0.71	0.01	1.01	9.03	0.47	1.06
2013:2	0.04	0.00	1.11	0.34	−0.02	0.95	1.18	−0.11	0.91	9.06	−2.00	0.82	0.40	−0.30	0.57	9.11	0.07	1.01
2013:3	0.04	0.00	1.09	0.44	0.10	1.28	1.32	0.15	1.13	9.01	−0.05	0.99	0.39	−0.02	0.96	9.49	0.39	1.04
2013:4	0.03	−0.01	0.76	0.76	0.31	1.71	1.32	−0.01	1.00	8.64	−0.37	0.96	0.28	−0.10	0.74	9.93	0.44	1.05
2014:1	0.04	0.01	1.19	0.91	0.15	1.20	1.12	−0.20	0.85	8.73	0.09	1.01	0.29	0.01	1.02	9.95	0.01	1.00
2014:2	0.03	0.00	0.89	0.99	0.08	1.09	1.01	−0.11	0.90	8.86	0.13	1.02	0.32	0.03	1.11	10.47	0.53	1.05
2014:3	0.03	0.00	1.00	0.96	−0.03	0.97	0.99	−0.02	0.98	8.76	−0.10	0.99	0.26	−0.06	0.81	10.98	0.51	1.05
2014:4	0.03	−0.01	0.85	0.81	−0.15	0.84	0.88	−0.11	0.89	8.26	−0.50	0.94	0.24	−0.02	0.92	12.02	1.04	1.09
2015:1	0.03	0.00	1.14	0.85	0.04	1.05	0.86	−0.02	0.98	8.54	0.28	1.03	0.21	−0.04	0.84	12.41	0.39	1.03
2015:2	0.03	0.00	1.03	0.91	0.06	1.07	0.89	0.03	1.03	9.04	0.50	1.06	0.20	0.00	1.00	12.45	0.04	1.00
2015:3	0.04	0.00	1.02	0.81	−0.10	0.89	0.85	−0.04	0.96	9.00	−0.05	0.99	0.16	−0.05	0.77	12.79	0.34	1.03
2015:4	0.03	−0.01	0.79	0.72	−0.09	0.89	0.83	−0.03	0.97	8.94	−0.05	0.99	0.14	−0.02	0.89	13.24	0.45	1.04
2016:1	0.03	0.01	1.20	0.60	−0.12	0.83	0.84	0.01	1.02	8.76	−0.18	0.98	0.16	0.02	1.11	14.13	0.88	1.07
2016:2	0.03	0.00	0.87	0.52	−0.08	0.87	0.82	−0.02	0.97	8.81	0.05	1.01	0.19	0.03	1.22	15.06	0.93	1.07
2016:3	0.03	0.00	1.00	0.51	−0.02	0.97	0.90	0.09	1.11	9.49	0.68	1.08	0.15	−0.04	0.81	16.24	1.18	1.08
2016:4	0.03	0.00	1.12	0.49	−0.02	0.96	0.87	−0.03	0.97	9.37	−0.13	0.99	0.14	−0.02	0.89	15.94	−0.30	0.98
2017:1	0.04	0.00	1.07	0.57	0.08	1.16	0.86	−0.01	0.99	10.25	0.88	1.09	0.15	0.01	1.10	16.41	0.47	1.03
2017:2	0.04	0.00	1.04	0.54	−0.03	0.95	0.88	0.01	1.02	10.53	0.28	1.03	0.16	0.01	1.04	16.11	−0.30	0.98
2017:3	0.04	0.01	1.17	0.54	0.00	1.00	0.90	0.02	1.02	11.62	1.09	1.10	0.15	0.00	1.00	16.86	0.76	1.05
